# Exploring transformative learning for trainee pharmacists through interprofessional simulation: a constructivist interview study

**DOI:** 10.1186/s41077-021-00180-2

**Published:** 2021-09-07

**Authors:** Victoria R. Tallentire, Joanne Kerins, Scott McColgan-Smith, Ailsa Power, Fiona Stewart, Julie Mardon

**Affiliations:** 1 NHS Education for Scotland, Scotland, UK; 2 Scottish Centre for Simulation and Clinical Human Factors, Larbert, NHS Forth Valley UK; 3 University of Edinburgh, Edinburgh, UK; 4 NHS Greater Glasgow and Clyde, Glasgow, UK; 5 NHS Ayrshire & Arran, Crosshouse, UK

**Keywords:** Interprofessional, Transformative, Pharmacy, Simulation

## Abstract

**Background:**

The expanding roles of UK pharmacists have prompted substantial changes to the initial pharmacy education and training, including increasing recognition of the value of learning alongside other professional groups in acute settings. Interprofessional immersive simulation training appears to represent a useful educational tool to meet the evolving needs of the profession, but the impact of such training on workplace behaviour and relationships has not been explored. This study aimed to explore how interprofessional simulation training facilitates transformative learning in pre-registration pharmacists.

**Methods:**

Across three different locations in Scotland, pre-registration pharmacists were paired with medical students to participate in immersive simulation scenarios with post-scenario debriefs. Pre-registration pharmacists were individually interviewed shortly after their simulation session, using a semi-structured interview schedule based on the transformative learning framework. Transcripts were analysed using template analysis, with Mezirow’s phases of perspective transformation forming the initial coding template.

**Results:**

Fifteen interviews following five simulation sessions at three different sites were undertaken. Phases 1–6 of the transformative learning framework all resonated with the pre-registration pharmacists to varying degrees. Two prominent threads became evident in the data: a change in participants’ perceptions of risk, and deepened understanding of their role within an acute context. These themes were woven throughout phases 2–6 of the transformative learning framework.

**Conclusions:**

Interprofessional immersive simulation training involving acute clinical scenarios has been found to be helpful for pre-registration pharmacists and can foster transformative learning. Through this powerful process, they developed new ways to see the world, themselves and their professional relationships. Positive future actions and roles were planned. As the patient-facing roles of pharmacists expand, educational practices that translate into meaningful change to workplace behaviour and relationships become increasingly important. Carefully constructed interprofessional immersive simulation training should be utilised within pharmacy education more widely.

**Supplementary Information:**

The online version contains supplementary material available at 10.1186/s41077-021-00180-2.

## Introduction

Whilst the use of simulation-based education (SBE) is firmly embedded within medical and nursing training in the United Kingdom (UK), its adoption into pharmacy training has been slower. The benefits of simulation in pharmacy training are widely recognised [1, 2], but there remains uncertainty as to how to ensure synergy with existing training and maximal impact on future practice [3]. Immersive simulation, one form of SBE, is designed to replicate substantial aspects of the real clinical environment in a fully interactive way, often with the aid of a full body high-fidelity mannequin simulator [4]. Such training can facilitate the development of skills and attitudes, particular in the domains of cognition and teamworking, in a controlled and safe way [1, 4].

The evolution of pharmacists’ roles within UK hospital settings over the last 15 years has necessitated the development of novel educational interventions [3]. Enhanced roles within emergency and acute medicine departments, attendance on ward rounds, leading outpatient clinics and prescribing mean that pharmacists of today require a complex, expanded skillset. This new skillset includes the need to work flexibly alongside other healthcare professionals, as well as patient consultation skills rooted in the principles of patient-centred care [3]. In recognition of this challenge, substantial changes to the initial education and training of pharmacists are currently being implemented in the UK by the General Pharmaceutical Council (GPhC) driven, in part, by the need for increasing emphasis on the development of skills in decision-making, communication and risk management [5]. As patient-facing roles of pharmacists within UK hospitals increase, so too does the likelihood of them being more involved in emergency situations, a context in which pharmacist input has been associated with reduced patient mortality and a reduced risk of adverse drug events [6, 7]. The use of immersive SBE has been shown to increase postgraduate year one pharmacy residents’ perceived competence in common medical emergencies in the USA [8, 9], but this aspect of training has received little attention in the UK. With the divers of developing an expanded skillset and confidence in the acute setting in mind, immersive SBE would appear to offer an opportunity worth exploring further within UK pharmacy training.

The expanded roles of pharmacists in the UK clinical workplace also necessitates a more prominent role for interprofessional education (IPE). According to the World Health Organization, IPE occurs when “students from two or more professions learn about, from and with each other to enable effective collaboration and improve health outcomes” [10]. The ability to replicate relevant experiences, address specific technical and non-technical learning outcomes and orchestrate useful conversations between various healthcare professionals positions immersive simulation as an educational tool ideally situated to deliver IPE [11]. Widely incorporated into UK medical and nursing curricula, IPE has been shown to have a positive impact in terms of attitudes, perceptions, collaborative knowledge and skills [12]. Within pharmacy education, implementation of sporadic teaching initiatives has shown interprofessional SBE to be relevant and valued and to increase confidence in communication with other healthcare professionals [1, 13]. However, it is unclear whether the positive experience of IPE within pharmacy education translates into meaningful change to workplace behaviour and relationships.

Transformative learning (TL) is learning that challenges established perspectives, leading to new beliefs and ways of being [14]. It is a complex metatheory that has become established across numerous disciplines in higher education, including the health professions [15, 16]. SBE can have significant and sustained effects on participants, and its potential to create a learning experience that is transformative has been recognised [17–21]. Previous work has examined the features of a simulated learning environment that contributed to transformative learning experiences [22], and the importance of emotional congruence in supporting transformative change [23]. Situated firmly within a interpretivist paradigm, TL is a theory of learning that delineates the ways in which our pre-existing assumptions, expectations and relationships influence the meaning derived from new experiences [24]. As a pedagogy that facilitates the recognition and incorporation of the complexities of the clinical workplace, TL is the ideal lens with which to explore the more nuanced and sustained effects of IPE [15, 25].

## Aim

The aim of this study is to explore the how interprofessional simulation training facilitates transformative learning in pre-registration pharmacists.

## Methods

Ethical approval for this study was obtained from NHS Education for Scotland Research Ethics Committee (approval no. NES/Res/13/20/Ph). All participants provided written consent to the collection and publication of anonymised results and were free to leave the study at any time, without reason or penalty.

### Conceptual framework

The theoretical construct of TL was first introduced through the work of Mezirow in 1991, who described ten phases of perspective transformation to describe the process of TL [14, 26]. The first phase is that of a “disorientating dilemma” which challenges an individual’s existing beliefs or assumptions. This has previously been accepted to align with the experience of participating in immersive simulation involving the assessment and treatment of an acutely unwell patient [17, 19]. The second phase relates to a process of self-examination followed by a critical assessment of assumptions (phase 3) involving questioning and reappraisal of previous presuppositions [27]. It is this change in meaning perspectives that is at the heart of TL—a world view shift [28]. Mezirow’s fourth phase, recognition that one’s discontent and process of transformation are shared, emphasises the strong social imperative for TL by presupposing engagement with others negotiating similar changes in perspective [15]. The development of a new perspective prompts exploration of options for new roles, relationships and action (phase 5). Although TL is characterised as having a prominent cognitive dimension, its impact should manifest as behavioural change as encapsulated in phase 6, planning a course of action. Phases 1–6 can therefore be encapsulated within a simulation training session involving an immersive scenario followed by subsequent debriefing which promotes reflection and provides an opportunity to question and discuss prior assumptions, the experiences of others and plans for the future [17].

The consequent phases of perspective transformation described by Mezirow are acquisition of knowledge and skills for implementing one’s plans (phase 7), provisionally trying out new roles (phase 8), building competence and self-confidence in new roles and relationships (phase 9) and reintegration into one’s life on the basis of conditions dictated by one’s new perspective (phase 10). These later phases are likely to be navigated following a return to the workplace. Therefore, phases 1–6 of perspective transformation, as described by Mezirow, provide the conceptual framework for this study.

### Study design

For this constructivist study, pre-registration pharmacists were paired with medical students to participate in immersive simulation scenarios. Post-scenario debriefs focussed on a range of technical and non-technical factors, including teamworking. Participants were individually interviewed shortly after their simulation session, using a semi-structured interview schedule based on the transformative learning framework [29]. Transcripts were analysed using template analysis, with Mezirow’s phases 1–6 of perspective transformation forming the initial coding template.

### Participant recruitment

In the UK, following completion of a GPhC-accredited pharmacy qualification, aspiring pharmacists complete the GPhC pharmacist pre-registration scheme consisting of a year-long training placement and a registration assessment [30]. From July 2021, pre-registration pharmacists will be known as trainee pharmacists undertaking their Foundation Training Year. Pre-registration pharmacists were recruited to this study on a voluntary basis through NHS Education for Scotland (NES). An email invitation was disseminated to all hospital-based pre-registration pharmacists in the West and North regions of Scotland, for half day simulation sessions taking place in NHS Ayrshire & Arran and NHS Highland respectively. Most pre-registration pharmacists working in Scotland are graduates of one of the two Scottish pharmacy schools located at the University of Strathclyde in Glasgow [31] and Robert Gordon University in Aberdeen [32]. At the time of this study, neither university course incorporated any immersive simulation training in their curricula, although such training exists elsewhere [33, 34]. At the time of recruitment, the pre-registration pharmacists were about half way through their pre-registration year and would all have shadowed specialist pharmacists in various areas of their respective hospitals (including some acute settings), but would be directly supervised and would not have been given sole responsibility for patients. All pre-registration pharmacist attendees were provided with information via email, in advance of their simulation session, relating to the content of the session, the purpose of the study and the optional post-session interview. Final year medical students were chosen to participate with pre-registration pharmacist in this study to avoid a perception of hierarchy between a group who had completed their qualification examinations and those who were yet to sit them. Furthermore, both groups are largely supernumerary in their respective clinical areas and could therefore attend a simulation session without significant patient care implications. Medical students were also recruited on a volunteer basis via their respective Universities, Glasgow and Aberdeen.

### Simulation session

Between December 2020 and March 2021, the half-day simulation session was delivered five times across three sites: University Hospital Ayr and University Hospital Crosshouse in Ayrshire, and the Centre for Health Science in Inverness. The overarching aim of the session was to allow both groups of learners to gain experience of working closely with individuals from different professional groups, within an acute care context. Each session was attended by three pre-registration pharmacists and three medical students, with one of each group participating in each scenario as a pair. Each immersive scenario had been carefully designed and piloted (with several groups of pre-registration pharmacists and medical students who were not study participants), to ensure a combination of medical and pharmaceutical learning outcomes, pitched at the appropriate level. Learning outcomes and scenarios were jointly authored by SMS and JM. The scenarios chosen for this study were those that performed most consistently in the pilot sessions: asthma exacerbation, urosepsis on a background of Parkinson’s disease and acute stroke with dysphagia.

Immersive simulation was used to provide the contextualised data for this study as an appropriately devised and delivered simulation scenario has previously been conceptualised as the relevant “trigger” experience for TL [17, 19–21]. The simulated environment consisted of a single, full-body adult mannequin simulator (SimMan Essential; Laerdal) accompanied by paperwork (including repeat and acute prescription details in the form of an Emergency Care Summary, prescribing and monitoring charts), monitoring equipment, drugs and other supplies as available in a standard emergency department. Three ceiling-mounted cameras allowed each scenario to be filmed from a variety of perspectives and relayed real-time to both the control room and non-participating attendees. The patient voice was transmitted via a wireless microphone and a bedside monitor displayed dynamic physiological parameters aligned with the clinical presentation. A member of staff, unknown to the participants, and capable of a pre-defined range of tasks, played the role of a nurse within each scenario. Each 15-min scenario was followed by a video-assisted debrief lasting approximately 40 min, during which discussion of the clinical and non-technical aspects of the case was facilitated by expert pharmacist (SMS) and medical (JM) simulation faculty. Debriefing was aided by immediate playback of the scenario and encouraged articulation of the cognitive processing occurring during the scenario.

### Interviews

Following their attendance at a simulation session, each consenting pre-registration pharmacist was contacted by email to arrange an individual interview. To comply with COVID-19 travel and social distancing requirements, all interviews were undertaken using Microsoft Teams. Individual interviews were chosen due to the ability to explore sensitive topics without the presence of peers and to facilitate deep reflection on individual feelings, assumptions and relationships [35, 36]. Interviews were performed by VT, a physician and experienced simulation facilitator and qualitative researcher who had not been involved in the delivery of the simulation session. Previously unknown to all participants, she was introduced as “a researcher” with the aim of encouraging uninhibited discussion by de-emphasising any perceived power imbalance [37]. A semi-structured approach was adopted, with initial questions based on Mezirow’s transformative learning framework [29], with questions evolving over time to allow deeper exploration of emerging themes. Interviews were audio-recorded, transcribed verbatim and anonymised. Data collection ceased when data saturation was reached, defined as the absence of new sub-themes and the inability to produce new codes [38].

### Analysis

After each batch of interviews, VT and JK, a physician and experienced medical education researcher, coded the data into the transformative learning framework using template analysis [39]. In template analysis, the initial coding framework, as well as providing a lens to illuminate and explore the data, may be modified by the data, with new codes added inductively [39]. When the meaning of a statement was unclear, reference was made to the original interview recording so that the presence of intonation or emphasis could aid interpretation. Analysis of early interviews commenced in parallel with continued data collection in order to facilitate the deeper exploration of emerging themes with subsequent participants. Following independent coding, VT and JK discussed each phase of the transformative learning framework in detail, to identify themes and their associated dimensions. Disagreement was resolved with reference to Mezirow’s original descriptions and subsequent analyses of his work [27, 29], with final decisions made by VT. Once defined, the emergent themes relating to each phase of Mezirow’s transformative learning framework were presented to SMS and JM, experienced clinicians and the facilitators of the simulation sessions, in order to ensure contextual validity and resonance with their facilitation experiences. In this constructivist study, the concept of an objective reality has been rejected and there is an explicit recognition that the framework produced is VT’s conceptualisation of the data produced by the interactions between VT, JK, their co-researchers and the participants.

## Results

Between December 2020 and March 2021, a total of 15 pre-registration pharmacists (PRPs) participated in five simulation sessions, with all 15 engaging in a subsequent online interview. Of those interviewed, nine had participated in University Hospital Crosshouse, three in University Hospital Ayr and three in the Centre for Health Science, Inverness. Interviewees were aged between 22 and 27, 14 identified as female and one identified as male. All interviews took place between three and 16 days following the respective simulation sessions (mean 7 days). Interviews lasted between 18 and 29 min (mean 23 min). Phases 1–6 of the transformative learning framework all resonated with the pre-registration pharmacists to varying degrees. A summary of the subthemes identified in relation to each of the two prominent threads of TL described below, with exemplar quotes, is shown in Table 1.

**Table 1:** Summary of the subthemes identified in relation to the two prominent threads of TL

Phase of perspective transformation	Subthemes and exemplar quotes			
**Phase 1: A disorientating dilemma**	“Well, it was a bit nerve-wracking, a bit scary... I thought I was going to be really out of my depth” [PRP10] “I was nervous, I didn’t really know what to expect.” [PRP9]			
	**Understanding of role**		**Perception of risk**	
**Phase 2: Self-examination with emotional disturbance**	**Value in an acute situation**	“I was quite apprehensive…I didn't really know what value I could add to that situation in such an acute setting.” [PRP12]	**Frustration at lack of information**	“I thought it was quite difficult… making decisions quickly and reading guidelines quickly and trying to find the best evidence and then relaying that.” [PRP9]
	**Pressure of concurrent assessment**	“I suppose normally I would go away and look at a patient myself before coming to a medic rather than do it both at the same time, almost like a car crash…. I felt quite stressed out to be honest. I came out in a bit of a cold sweat.” [PRP5]	**Concern about causing harm**	“..there wasn’t a diagnosis, the bloods weren’t there.. all the stuff that I would normally look at before recommending any interventions… if we missed something in the diagnosis, if we missed something, like, in such an acute setting.” [PRP10]
**Phase 3: A critical assessment of assumptions**	**Transferrable skills**	“You’re working directly together, communicating constantly… very rapidly changing your plan of action and communicating that to people and coming up with an idea of what you’re going to do next and delegating small tasks to each other...” [PRP8]	**Decisions made with incomplete information**	“I like knowing the answer and having a specific answer, but I am aware that’s not always the case and it’s kind of risk and benefit, judging it. And so it was putting me out of my comfort zone in that sense because there is no right or wrong answer… not everything’s going to be 100 per cent safe, but it’s weighing it up.” [PRP1]
	**Problem-solving**	“I have never really had that problem- solving part in my job really, usually they have already been diagnosed by the time I've seen them, so it really kind of switched me back into that kind of helping to diagnose them as well, which was nice.” [PRP6]	**Prioritisation of urgent issues**	“It was interesting to, sort of, consider what is a problem that we needed to solve then, and what would be a problem that would be further downstream in the wards… I did find I had to really balance that and think, no, that’s a future problem. That’s not something we’re sorting right this second.” [PRP3]
**Phase 4: Recognition that one’s discontent and process of transformation are shared**	**Value realised by others**	“I think every single time I’ve had interaction with, particularly medics, it’s felt as though, the biggest surprise to them is how much I can offer in terms of helping them, with these kind of situations.” [PRP8]	**Self-doubt and fear of causing harm**	“I think we were both kind of, a bit worried by it.. I think we shared the same kind of nerves for it… during the actual scenario, I thought he [medical student] was just getting on with everything and I didn’t really notice the nerves drumming in that situation, it was just when we discussed it afterwards.” [PRP10]
**Phase 5: Exploration of options for new roles, relationships and actions**	**Growing in confidence as a pharmacist**	“I did feel like I was making decisions, which I feel like is what a pharmacist does… I think I actually felt as close to a pharmacist as I’ve felt.” [PRP5]	**Sharing knowledge**	“The knowledge, kind of, just came back to me, if that makes sense. I just wasn’t really thinking about it. I was just, kind of, doing it… I think the, sort of, working together part helped with that. If I was making a quicker decision, working with someone else who’s also a different profession made you feel a bit more comfortable in your decisions.” [PRP4]
	**Value in the team**	“More towards the end, the doctor was like ‘is there anything I can do to help you?’ I suppose it made me realise that it is that team, you are just one small part of trying to fix this problem for the patient.” [PRP6]		
	**Shared decision-making**	“So, it’s good to know that we’re all, kind of, on the same page and actually that’s why it’s so important to make decisions together.” [PRP3]		
**Phase 6: Planning a course of action**	**Approaching medical staff**	“…being more comfortable communicating with the doctors and just, maybe if they don’t ask me questions, if they’re not too sure what my role is, I could maybe, do you know, speak to them… So, if I’m going in to speak to them, I could tell them what I’ve done and what I’ve looked up and, yeah, just building relationships with the doctors.” [PRP9]	**Prioritising information**	“I could probably find quite a lot of information, but it’s not always as easy for the medics to do that, and they may not need all the information that I’ve found. So, it’s just trying to then approach it, and give them the concise information.” [PRP1]
	**Education of medical staff**	“I suppose not seeing doctors as, they don't know everything… they might be like the boss of the ward but they still make mistakes, so not to be afraid to call them out on the mistakes and help them.” [PRP7]	**Speaking up**	“I think maybe [I will] be more vocal when I think that input is needed, because I was able to see quite clearly how the knowledge that I could bring to the scenario changed the path that it went down… I think speaking up and actioning things and then maybe just taking on more of a role and responsibility if there’s something that needs to be done, just being like ‘oh I’m from pharmacy, I can do that’.” [PRP10]
	**Building professional relationships more generally**	“I’d definitely be quite confident with speaking to doctors, speaking to other health professionals about things I wasn’t sure of, as well as other pharmacists, just everyone really, if I wasn’t sure about something. And I’d have maybe a slightly better understanding of what they know that I may not.” [PRP4]		

### Phase 1: A disorientating dilemma

The first phase of perspective transformation involves challenge of an individual’s existing beliefs or assumptions. The PRPs described feeling “chucked in the deep end” [PRP2] and “out of my comfort zone” [PRP1], descriptions that resonate with the “disparate experience” described by Mezirow [40]. An element of fear is often characteristic of such experiences [15], evident in the PRPs descriptions of feeling “kind of frightened” [PRP2] and “quite stressed out to be honest” [PRP5].

### Phase 2: Self-examination with emotional disturbance

Mezirow’s initial framework emphasised the presence of guilt and shame in the second phase of perspective transformation [29]. A wider range of emotions seemed to be experienced by the PRPs who also expressed frustration and concern when reflecting on their performances. At this stage, the two prominent threads of TL became evident in the data: self-examination in relation to the PRP’s *perception of risk*, and the same process in relation to an *understanding of their role* within an acute context.

Frustration stemmed from the realisation that medical practice sometimes differed from the theoretical assumptions on which their education had largely been based:

It’s a bit frustrating because they do teach you to get as much information as you can… I felt very stressed and I had to try my best to stay calm, because if you don’t stay calm you’ll just not achieve anything. [PRP2]

A sense of responsibility gave rise to the realisation that there was the potential to cause harm:

It made me feel quite uneasy, because it was not knowing the outcome and not having all the information. And then having this concern, is my decision going to have a negative impact? [PRP1]

There was a degree of concern about being involved at such an early stage of the patient journey, and a fear that “there wouldn’t be a role for me in that kind of emergency situation” [PRP5] resulting in them “feel[ing] a wee bit useless” [PRP10] or being perceived as a “dead weight” [PRP15].

The PRPs felt that making rapid decisions, sometimes with incomplete information, was extremely stressful and they had particular concerns relating to when and how to share information that they had not had time to check:

It was quite difficult… making decisions quickly and reading guidelines quickly and trying to find the best evidence and then relaying that… normally, I have a lot of time to think about what I’m going to say before I say it to someone else. [PRP9]

### Phase 3: A critical assessment of assumptions

The emotions experienced during self-examination led the PRPs to reflect on their assumptions relating to both their perception of risk and their role*.*

The challenge of being involved in the management of an acutely unwell patient resulted in a re-examination of the belief that all medication decisions require complete information: “I was trying to figure out the balance between acting in a timely manner but having the complete information” [PRP1].

There was a shift from trying to elicit all relevant information to trying to glean essential information, requiring a degree of prioritisation that was unfamiliar:

It made me step back and think is this crucial information that I need right this minute, is it urgent that I know this information… so you just kind of make it brief and get the information you need to know. [PRP2]

The requirement to make decisions quickly in a high-pressure context prompted the PRPs to reflect on their current skillset, particularly in relation to their teamworking skills that were more transferrable and relevant than they had appreciated:

I think there’s certain times in pharmacy where you do get put under quite a lot of pressure… it’s still very transferrable to other kind of pressures that I have to deal with and being able to find kind of, ways of coping with that pressure through team work and utilising the other members of the team that are available to you. [PRP8]

There was a realisation that pharmacist involvement does not have to wait until a diagnosis has been reached and that working alongside a medic could lead to more stream-lined problem-solving:

I realised that if I wasn’t there, I think it wouldn’t have went as smoothly. I think the doctor was appreciative that I was able to go and find the guidelines and know… I knew about the antibiotics… So, I was able to do that while he was able to do other things, so I think it did save time… I was, kind of, appreciated being in the room. [PRP9]

### Phase 4: Recognition that one’s discontent and process of transformation are shared

The debrief that followed the immersive simulation allowed the PRPs to hear each other’s perspectives and appreciate that their emotional disturbance and process of transformation were shared by both their PRP colleagues and the medical students.

Regarding perception of risk, the PRPs realised that the medical students not only shared their frustrations, but also their self-doubt and concerns relating to error and potential harm:

I’ve probably always thought they [medical students] were quite sure of themselves. And actually, it was seeing their reflections afterwards… just seeing that and realising that, actually, they’re going through the same sort of decision-making processes as we are, and they have the same sorts of doubts. [PRP3]

The PRPs were able to recognise the process of transformation in the medical students, particularly in relation to the added value that a pharmacist could bring to an acute situation. This helped to legitimise the PRP’s evolving understanding of their role.

it’s made them [medical students] realise how pharmacists can help on the ward… I feel like they do appreciate what we can do to help them. So hopefully their views have changed on what pharmacists do. [PRP2]

### Phase 5: Exploration of options for new roles, relationships and actions

As the PRPs developed new perspectives, their worldviews shifted. This process occurred in relation to both threads of TL.

In terms of perception of risk, the PRPs began to appreciate that they had useful knowledge that could, in certain acute situations, contribute in important ways to a good patient outcome. At such times they needed to speak out and share their knowledge in succinct and helpful ways, even in the absence of total certainty. This would previously have been regarded by the PRPs as risky or even cavalier behaviour, but their evolving perception of risk—as something that cannot be eliminated entirely within acute medical situations—made them more likely to share knowledge or ideas without total certainty.

…knowing when to sort of jump in with my knowledge and stuff, knowing when to step in and give the information to the doctor at a certain time, knowing what information that you have is useful, and what is maybe less helpful, and then what you can do, moving on from that, to help support the care that the doctor is trying to give. I think now I understand that a lot better. [PRP12]

TL influenced how the PRPs viewed themselves as professionals, encouraging a process of self-actualisation that seemed to build self-confidence and empowerment:

I think walking out of it I felt like a pharmacist, but I didn’t feel like that walking in. Not at all…. But actually, feeling like a pharmacist, that definitely was a feeling, we’re now beginning to get more and more glimpses of maybe being a pharmacist one day… I think it was genuine. [PRP3]

Through the process of TL, the PRPs reflected on their *role within the healthcare team*, particularly in the context of managing an acutely unwell patient.

It makes me more aware what everyone brings to the team, if that makes sense, your role in an MDT team. And what you might know and what you might not know…it’s made me a lot more aware of that, I think it’ll be easier to work together with other professionals in the future. [PRP4]

Maturation of professional identity and increased self-confidence led to a subtle reconceptualisation of the relationship between PRPs and medical students and, by extension, between pharmacists and doctors. A relationship that had been perceived as transactional and regulatory evolved into one characterised by shared decision-making and mutual respect.

To see somebody [medical student] turn round and say, actually, ‘Is there anything else you need, so that you can make these decisions?’ …I don’t know, that just, to me, worked really well. [PRP3]

### Phase 6: Planning a course of action

Despite the prominent cognitive dimension of TL, tangible behavioural change is an important feature [15]. The PRPs were keen to act on the insights derived from their new perspectives and reconceptualised relationships. In response to their enhanced understanding of the *balance of risks* in an acute situation, the PRPs planned to employ more refined communication strategies in emergencies, aware of the need to prioritise information and be concise:

I like to have all the information and say something really thoroughly, [but] I’m trying to communicate with someone who’s got a lot on their mind and a lot of priorities, then it’s, kind of, keeping it concise. And thinking ‘what are the main points they actually need to know?’ …and then obviously understanding what their priorities are, so I can help tailor whatever advice or suggestions they need. [PRP1]

The PRPs also planned to change their communication styles with medical staff more generally, feeling more confident to speak up in acute situations, as well as approach doctors and offer their assistance or advice. PRPs reflected that during previous approaches to medical staff they had often seemed “apologetic” [PRP3], but they planned in the future to be “more assertive” [PRP3] and “less apologetic and less assumptive when I am coming forward with a medication issue… if it was something they [medical staff] weren’t too sure about, and I had the knowledge, why wouldn’t I share that and then the next time they came across it, they would know what to do? So, I think going forward, I would probably try and do a bit more of that” [PRP5].

Through gaining an insight into the fallibility of doctors, PRPs reconceptualised both their *own roles and relationships with medical staff*, moving from “pharmacy police who just check their work” [PRP6] to trusted, confident colleagues and educators.

I think it would make me more likely to speak up rather than just assume that they [doctors] would know… Sometimes I don’t really know how much to give them because I don’t want them to think that I am condescending. I don’t want them to think, ‘well obviously I know that, why is she telling me that? She is wasting my time’. But it would probably make me more likely to speak up with things and then, even if they did know, I suppose, it’s better to give them the information than not. [PRP5]

The PRPs’ transformed views of themselves as valued and trusted members of the healthcare team seemed to extend beyond their relationships with medical staff and influence plans to invest more generally in their professional relationships:

I think sometimes it just depends on who you’re working with, but on an individual basis you need to make the effort to try and communicate with the person. I feel like if you make the first step, communication can run a lot smoother… it’s made me realise that communicating with someone is much better than not saying anything at all. So, I feel that I would be fine now to talk to anyone in the hospital about anything. [PRP2]

## Discussion

This study used the phases of perspective transformation described by Mezirow as a lens through which to explore the transformative learning experienced by PRPs engaging in interprofessional simulation training. Immersive simulation, with its emphasis on guided reflection on a concrete experience, is ideally constructed to initiate the process of perspective transformation. Whilst the positive effects of IPE within pharmacy education have been studied [1, 13], the extent to which such training may challenge preconceived understandings, enable positive future action and foster meaningful relationships was not well understood. This study has added to the literature by delineating the emotional, cognitive and social dimensions of the TL experience in the context of interprofessional SBE.

The immersive simulation scenarios provided the conditions to “trigger” TL for the PRPs, and the associated emotional disturbance that initiated their perspective transformation included frustration and fear. The disorienting dilemma, on which TL relies for initiation, is of particular interest to simulation facilitators. In an educational setting, such an experience needs to be carefully structured “to encourage the learner to commence the TL journey” [41]. The necessary affective component of the disorientating dilemma is, however, at odds with traditional immersive simulation rhetoric emphasising “safe spaces” and the avoidance of heightened emotion which can hamper performance [42]. Whilst such conflicting educational paradigms have been previously discussed [17], there is limited understanding of how unsettling simulated experiences can be better utilised to initiate TL whilst avoiding unresolved learner distress or “forced” transformation being perceived as indoctrination [41].

The most powerful results of this study relate to the ways in which PRPs reconceptualised their own roles and the relationship between PRPs and medical students. By extension, the exploration of new roles and plans for future action revealed a corresponding reframing of the relationship between pharmacists and doctors. In turn, this led to a newfound sense of confidence in their interactions with medical staff and plans to be more assertive and involved. Whilst empowerment is a recognised and celebrated outcome of TL, it must be remembered that any resultant shifts in relationships and power dynamics will be situated within the complex reality of the clinical workplace. There are many practical, cultural and attitudinal differences between the professions of medicine and pharmacy that influence relationships and patient care [43]. Although it is recognised that any intended behavioural changes could be jeopardised by longstanding interprofessional tensions and pervasive hierarchies [44, 45], the PRPs’ plans to form more collaborative, symbiotic partnerships with doctors is uplifting.

The second prominent thread evident in the results, that of an evolving perception of risk, is also of particular interest in light of the proposed changes to UK pharmacy education [46]. The PRPs commenced the interprofessional SBE with a unidimensional conceptual model of risk as an entity to be minimised through the acquisition of sufficient information and considered decision-making. The cautious, risk averse behaviours of pharmacists have been well-documented [47, 48], and are strengths in the majority of contexts in which they work. However, through the process of perspective transformation, exposure to acute situations prompted the PRPs to reconceptualise risk as a dynamic phenomenon, characterised by tensions between additional information and rapid treatment, and where direct harm from the actions of the healthcare team becomes ethically indistinguishable from harm due to omission or delay [49]. The results of this study show that interprofessional SBE, through the power of TL, may challenge some of the assumptions and ingrained characteristics of pharmacists, including “lack of confidence, fear of new responsibilities, paralysis in the face of ambiguity, need for approval and risk aversion” [50] that may represent barriers to change within the profession [51].

### Strengths and limitations

This study was conducted across three centres in Scotland, allowing involvement of a range of PRPs who worked in various Health Boards. The constructivist nature of the work means that ideas were co-constructed by the lead researcher, her co-researchers and the participants. Data collection and analysis will have been influenced by VT’s prior clinical and educational experiences, including her interactions with pharmacists. The results of this study may be transferrable to other contexts, but they are not generalisable. It is acknowledged that in addition to the simulation scenario and debrief, the interview process itself may have been contributed to the transformative learning process, through the articulation of cognitive processes that might otherwise have remained preconscious. Furthermore, some of the quotes suggest that, at time, some of the pre-registration pharmacists may have conflated the roles of medical students and doctors, making reconceptualisations of the relationships between PRPs and medical students, and by extension, pharmacists and doctors, more difficult to interpret in a consistent way.

Whilst the affective, social and behavioural facets of TL have ensured that it has become widely recognised as a desirable outcome for health professions education [15], criticisms of the theory, and its application, remain. The fundamental question of when a learning experience qualifies as “transformative” remains unanswered; much learning goes beyond the simple transfer of information, but the criteria that define TL remain largely opaque and unchallenged [15, 52]. Whilst presenting evidence of phases 1–6 of perspective transformation, this study does not include evidence of permanent behavioural change, as encapsulated by phases 7–10. In order to present evidence of all phases of perspective transformation, culminating in integrated and sustained behavioural change, a longitudinal study, with data collection at various stages, would be required [53]. However, it becomes difficult to attribute any long-term change observed in such studies to a single educational event.

### Future work

This study explored the perspective transformation of PRPs through interprofessional simulation training. Future work could examine the impact on medical student participants, whose identities, relationships and plans may have been influenced in similar ways. A longitudinal study involving interviews with the same PRPs as they transition to fully registered pharmacists might help to elucidate evidence of integrated behavioural change and illuminate some of the contextual factors that may inhibit transfer of training from a simulated environment to the clinical workplace. Audio diaries may be a useful adjunct to interviews, allowing participants to reflect on workplace encounters and relationships “in real time” and without the pressure of an interview [54]. In terms of educational practice, the results of this study strongly support the inclusion of interprofessional SBE for trainee pharmacists undertaking their Foundation Training Year, ideally within a broader framework emphasising acquisition of the communication, teamworking and shared decision-making skills that promote effective interprofessional working.

## Conclusions

Interprofessional immersive simulation training can foster transformative learning in PRPs. Through experiencing an emotionally disturbing event and reflecting on preconceived assumptions and understanding, PRPs developed new ways to see the world, themselves and their professional relationships. Positive future actions and roles were planned. As the patient-facing roles of pharmacists expand, educational practices that translate into meaningful change to workplace behaviour and relationships become increasingly important. This study lends weight to the argument for carefully constructed interprofessional SBE to be embedded within pharmacy education more widely.

## Supplementary Information

**Additional file 1.**

**Additional file 2.**

**Additional file 3.**

**Additional file 4.**

## Data Availability

The dataset generated and analysed during the current study are not publicly available as individual privacy would be compromised, but are available from the corresponding author on reasonable request.

## Abbreviations

SBE: Simulation-based education
UK: United Kingdom
GPhC: General Pharmaceutical Council
IPE: Interprofessional education
TL: Transformative learning
NES: NHS Education for Scotland
PRP: Pre-registration pharmacist

## References

[1] Regan K, Harney L, Goodhand K, Strath A, Vosper H (2014). Pharmacy simulation: a Scottish, student-led perspective with lessons for the UK and beyond. Pharmacy..

[2] Seybert AL, Smithburger PL, Benedict NJ, Kobulinsky LR, Kane-Gill SL, Coons JC (2019). Evidence for simulation in pharmacy education. JACCP.

[3] Lloyd M, Watmough S, Bennett N (2018). Simulation based training: applications in clinical pharmacy. Clin Pharm.

[4] Lateef F (2010). Simulation-based learning: Just like the real thing. J Emerg Trauma Shock.

[5] Reforms to initial education and training of pharmacists. An update from the Chief Pharmaceutical Officers and UK Pharmacy Regulators, 28 July 2020. 2020. Available from: https://www.pharmacyregulation.org/sites/default/files/document/joint_letter_from_cphos_and_uk_pharmacy_regulators_28_july_2020.pdf. Accessed 4 Apr 2021.

[6] Draper HM, Eppert JA (2008). Association of Pharmacist Presence on Compliance with Advanced Cardiac Life Support Guidelines During In-Hospital Cardiac Arrest. Ann Pharmacother.

[7] Bond CA, Raehl CL, Franke T (1999). Clinical pharmacy services and hospital mortality rates. Pharmacotherapy.

[8] Morris A, Young G, Roller L, Li F, Takamoto P, Baumgartner L (2019). High-fidelity simulation increases pharmacy resident perceived competence during medical emergencies. Curr Pharm Teach Learn.

[9] Thompson Bastin ML, Cook AM, Flannery AH (2017). Use of simulation training to prepare pharmacy residents for medical emergencies. Am J Health Syst Pharm.

[10] World Health Organisation. Framework for action on interprofessional education and collaborative practice. 2010. Available from: https://www.who.int/hrh/resources/framework_action/en/. Accessed 11 Apr 2021. 21174039

[11] West C, Graham L, Palmer RT, Miller MF, Thayer EK, Stuber ML, Awdishu L, Umoren RA, Wamsley MA, Nelson EA, Joo PA, Tysinger JW, George P, Carney PA (2016). Implementation of interprofessional education (IPE) in 16 U.S. medical schools: Common practices, barriers and facilitators. J Interprofessional Educ Pract.

[12] Guraya SY, Barr H (2018). The effectiveness of interprofessional education in healthcare: a systematic review and meta-analysis. Kaohsiung J Med Sci.

[13] Kayyali R, Harrap N, Albayaty A, Savickas V, Hammell J, Hyatt F, Elliott K, Richardson S (2019). Simulation in pharmacy education to enhance interprofessional education. Int J Pharm Pract.

[14] Mezirow J (1991). Transformative dimensions of adult learning.

[15] Van Schalkwyk SC, Hafler J, Brewer TF, Maley MA, Margolis C, McNamee L (2019). Transformative learning as pedagogy for the health professions: a scoping review. Med Educ.

[16] Lonie JM, Desai KR (2015). Using transformative learning theory to develop metacognitive and self-reflective skills in pharmacy students: a primer for pharmacy educators. Currents in Pharmacy Teaching and Learning.

[17] Kerins J, Smith SE, Phillips EC, Clarke B, Hamilton AL, Tallentire VR (2020). Exploring transformative learning when developing medical students' non-technical skills. Med Educ.

[18] McGaghie WC, Issenberg SB, Barsuk JH, Wayne DB (2014). A critical review of simulation-based mastery learning with translational outcomes. Med Educ.

[19] Bearman M, Greenhill J, Nestel D (2019). The power of simulation: a large-scale narrative analysis of learners' experiences. Med Educ.

[20] Parker B, Myrick F (2010). Transformative learning as a context for human patient simulation. J Nurs Educ.

[21] Smith KV, Witt J, Klaassen J, Zimmerman C, Cheng A-L (2012). High-fidelity simulation and legal/ethical concepts: a transformational learning experience. Nurs Ethics.

[22] Young JE, Williamson MI, Egan TG (2016). Students’ reflections on the relationships between safe learning environments, learning challenge and positive experiences of learning in a simulated GP clinic. Adv Health Sci Educ Theory Pract.

[23] O'Callaghan A (2013). Emotional congruence in learning and health encounters in medicine: addressing an aspect of the hidden curriculum. Adv Health Sci Educ.

[24] Taylor E, Merriam S (2008). Transformative learning theory. New Directions for Adult and Continuing Education.

[25] Zodpey S, Sharma A (2014). Advancing reforms agenda for health professionals' education through transformative learning. Indian J Public Health.

[26] Mezirow J, Welton M (1995). Transformation theory of adult learning. In Defense of the Lifeworld: Critical Perspectives on Adult Learning.

[27] An Overview on Transformative Learning in Ed. Illeris Knud: In: Illeris K, editor. 2nd edition. Contemporary Theories of Learning. Routledge; 2009:90–105. ISBN-10 : 1138550493. ISBN-13 : 978-1138550490.

[28] Kerins J, Tallentire VR. ‘Promises turn to dust…Trust into mistrust’: Medical students' experiences of mistreatment. Med Educ. 2021;55:423–5. 10.1111/medu.14440. 10.1111/medu.14440 33349951

[29] Mezirow J, Mezirow JA (1990). How critical reflection triggers transformative learning. Fostering Critical Reflection: A Guide to Transformative and Emancipatory Learning.

[30] Pharmacist pre-registration training scheme. GPhC; 2021. Available from: https://www.pharmacyregulation.org/education/pharmacist-pre-registration-training-scheme. Accessed 3 Apr 2021.

[31] University of Strathclyde G. Pharmacy. 2021. Available from: https://www.strath.ac.uk/courses/undergraduate/pharmacy/. Accessed 3 Apr 2021.

[32] Robert Gordon University A. Pharmacy. 2021. Available from: https://www.rgu.ac.uk/study/courses/883-mpharm-pharmacy. Accessed 3 Apr 2021.

[33] Thompson J, White S, Chapman S (2020). Virtual patients as a tool for training pre-registration pharmacists and increasing their preparedness to practice: a qualitative study. PLoS One.

[34] Cavaco AM, Madeira F (2012). European pharmacy students’ experience with virtual patient technology. Am J Pharm Educ.

[35] Rowley J (2012). Conducting research interviews. Manag Res Rev.

[36] Reeves S, Lewin S, Zwarenstein M (2006). Using qualitative interviews within medical education research: why we must raise the ‘quality bar’. Med Educ Oxford.

[37] Limerick B, Burgess-Limerick T, Grace M (1996). The politics of interviewing: power relations and accepting the gift. Int J Qual Stud Educ.

[38] Saunders B, Sim J, Kingstone T, Baker S, Waterfield J, Bartlam B, Burroughs H, Jinks C (2018). Saturation in qualitative research: exploring its conceptualization and operationalization. Qual Quant.

[39] King N, Symon G, Cassell C (1998). Template analysis. Qualitative methods and analysis in organizational research: a practical guide.

[40] An overview on transformative learning. 2nd edition. New York: Routledge; 2009:90-105. ISBN-10 : 1138550493. ISBN-13 : 978-1138550490.

[41] Saxena A (2019). Transformative learning: premise, promise and challenges. Med Educ.

[42] Fraser K, Ma I, Teteris E, Baxter H, Wright B, McLaughlin K (2012). Emotion, cognitive load and learning outcomes during simulation training. Med Educ.

[43] Gallagher RM, Gallagher HC (2012). Improving the working relationship between doctors and pharmacists: is inter-professional education the answer?. Adv Health Sci Educ.

[44] Gregory PAM, Austin Z (2016). Trust in interprofessional collaboration: perspectives of pharmacists and physicians. Can Pharm J.

[45] Rabani R, Key M, Morrissey H, Ball P (2021). Exploring students’ perceptions and opinions about an institutional hierarchy of healthcare professionals and its impact on their inter-professional learning outcomes. Pharm Educ.

[46] New standards for initial education and training of pharmacists approved General Pharmaceutical Council. 2020. Available from: https://www.pharmacyregulation.org/news/new-standards-initial-education-and-training-pharmacists-approved. Accessed 4 Apr 2021.

[47] McIntosh T, Munro K, McLay J, Stewart D (2012). A cross sectional survey of the views of newly registered pharmacists in Great Britain on their potential prescribing role: a cautious approach. Br J Clin Pharmacol.

[48] Cox A. We pharmacists are risk-averse but it is logical that we should all independently prescribe on registration. Pharm J. 2020;305. Available at: https://pharmaceutical-journal.com/article/opinion/we-pharmacists-are-risk-averse-but-it-is-logical-that-we-should-allindependently-prescribe-on-registration. Accessed 11 Apr 2021.

[49] Tallentire VR, Smith SE, Skinner J, Cameron HS (2011). Understanding the behaviour of newly qualified doctors in acute care contexts. Med Educ.

[50] Rosenthal M, Austin Z, Tsuyuki RT (2010). Are Pharmacists the Ultimate Barrier to Pharmacy Practice Change?. Can Pharm J.

[51] Rosenthal MM, Austin Z, Tsuyuki RT (2016). Barriers to pharmacy practice change: Is it our nature or nurture?. Can Pharm J.

[52] Newman M (2010). Calling Transformative Learning Into Question: Some Mutinous Thoughts. Adult Educ Q.

[53] Hoggan CD (2015). Transformative Learning as a Metatheory: Definition, Criteria, and Typology. Adult Educ Q.

[54] Monrouxe LV (2009). Solicited audio diaries in longitudinal narrative research: a view from inside. Qual Res.

