# Supplementary material for: Exploring transformative learning for trainee pharmacists through interprofessional simulation: a constructivist interview study
Source: Adv Simul (Lond). 2021 Sep 7;6:31. doi: 10.1186/s41077-021-00180-2 (PMC8422059; doi:10.1186/s41077-021-00180-2)
Supplement: Supplementary file 1 — Additional file 1. [file 41077_2021_180_MOESM1_ESM.docx]

**Semi-structured interview schedule for post-course interviews with pre-registration pharmacists**

**Introduction**

- Explain purpose of interview
- Highlight option to terminate at any time and withdraw from the study without giving a reason
- Confirm happy for audio recording and explain anonymity process
- General reflection on the course
  - *What did you think of the interprofessional simulation course you participated in on XXX?*
  - *How did you find the course?*
  - *What did you take away from it?*

**Specific topics**

- - *Did anything about the course challenge any of your beliefs/assumptions?*
  - *(If yes) How did that make you feel?*
  - *Did it make you re-evaluate what you believed?*
  - *Do you think that others on the course felt similarly (or differently)?*
  - *Do you think the feelings differed between the pre-reg pharmacists and the medical students?*
  - *Have you thought much about (the specific situation) since then?*
  - *Has this changed what you will do or how you will act in the future?*
  - *(If yes) In what ways?*
  - *Do you think this will change your relationships within the clinical workplace? (with medical students / doctors?)*
  - *Have you planned to take this learning further in any way?*
  - *Where would you like to go next with this?*

**Close**

- Is there anything else you would like to add to what has been said?
- Thanks and wrap up
