# Supplementary material for: Exploring transformative learning for trainee pharmacists through interprofessional simulation: a constructivist interview study
Source: Adv Simul (Lond). 2021 Sep 7;6:31. doi: 10.1186/s41077-021-00180-2 (PMC8422059; doi:10.1186/s41077-021-00180-2)
Supplement: Supplementary file 2 — Additional file 2. [file 41077_2021_180_MOESM2_ESM.docx]

**Acute Stroke in Elderly Gentleman**

**OUTLINE OF SESSION**

***Target Audience***

5^th^ Year Medical Student & Pre-registration/Junior Pharmacist.

***Synopsis***

Andrew Robertson, a 74 year old male, is brought in by ambulance with a ‘funny turn’. Family member following behind.

***Learning Objectives***

- Demonstrate team working skills and understand the different roles within the team
- Demonstrate appropriate communication skills with members of the multidisciplinary team
- Demonstrate an understanding of the immediate management of acute stroke
- Demonstrate an understanding of medication rationalisation in patient who is nil by mouth

***Environment, Equipment & Essential Props***

- Simulation room
- Mannequin (cannula insertion)
- 2+ faculty members
- ED yellow sheet
- Emergency Care Summary (ECS) Medicines Reconciliation Report
- Laptop with access to AthenA (stroke guidelines, sliding scale guidelines) and NEWT guidelines
- Fluid prescription chart
- High risk infusion chart
- ECG result
- CXR result
- Blood results
- NEWS Observation chart
- Nursing notes (initial swallow assessment will be documented there)

***Patient set up:*** in bed, monitoring attached

***Other participants:*** family member for history of presenting complaint and clarification of medicines

**SCENARIO SCRIPT**

**Background information**

Documented in notes - -patient experienced a ‘funny turn’ and is now not communicating very well. New left sided weakness. Family not present yet.

**Guidance for faculty (key aims)**

- Recognise acute stroke symptoms
- Request CT scan and refer patient to stroke consultant
- Recognise need to rationalise medicines and prescribe essential medicines via alternative route of administration
- Recognise need to withhold anticoagulant

**Predicted derails** (things which might not go according to plan)

- Does not identify acute stroke – nurse prompt
- Does not identify patient is nil by mouth – nurse prompt to nursing notes
- Does not recognise need to give some medicines by alternative route – nurse prompt to ECS
- Does not recognise poor compliance with warfarin – nurse to prompt blood results (INR)
- Does not clarify history of presenting complaint/medicines – family member prompt

**SCENARIO STORYBOARD**

| State | Events | Desired learner behaviours and trigger to move to next state | | | |
| --- | --- | --- | --- | --- | --- |
|  |  | Medic actions | Pharmacist actions | Mannequin operator | Teaching points |
| 1 | A: maintained  B: RR 18, O_2_ sats 99% on air  C: HR 125, BP 145/89  D: Temp 37.0^o^C  E: Abdo SNT | Don PPE  ABCDE assessment  IV access, bloods, fluids  +/- call for help | Don PPE  Medicines reconciliation  Recommend IV fluid options if asked | Patient unable to give history  ECG AF | Conduct ABCDE assessment  Task delegation |
| 2 | A: maintained  B: RR 19, O_2_ sats 99% on air  C: HR 127, BP 158/93  D: Temp 37.0^o^C  Left sided weakness | Neurological exam  Request CT scan  Ask nurse to request SLT assessment | Review/rationalise medicines as patient NBM  Recommend alternative route for anticonsulvants  +/- recommend IV digoxin | ECG AF  Expression discomfort on movement of left side | Importance of rationalising medicines  Switching medicines to alternative route of administration  Communication with the MDT |
| 3 | A: maintained  B: RR 19, O_2_ sats 99% on air  C HR 127, BP 157/91  GR 15.5 | Reassess  Recognise and consider management of hyperglycaemia | Refer to sliding scale guideline if asked  +/- assist with prescribing on high risk infusion chart | ECG AF | Referral to senior if needed |
| 4 | Family member arrives  HPC; sudden onset left sided weakness and aphasia. Usually fit and well.  DH: missed a few doses of warfarin as ran out of tablets and forgot to re-order | Confirm history of presenting complaint | Confirm medicines with family. Recognise non-compliance with anticoagulant could have led to stroke | Patient happy to see family member  ECG AF | Communication skills |

**Results**

Bloods:

Hb 144 g/L (133-176), WCC 8.0 x 10^9^/L (3.7-9.5), U 6.9 mmol/L (2.5-7.8), Cr 89 µmol/L (50-120), Na 135 mmol/L (133-146), K 4.4 mmol/L (3.5-5.3), LFTs normal, INR 1.3 (target 2.5).

CXR: clear lung fields, no consolidation, no pneumothorax.

ECG: ECG fast AF

**Expected Prescription**

- Discontinue ibuprofen, withhold warfarin
- Prescribe sodium chloride 0.9% IV
- Prescribe levetiracetam 750mg IV STAT
- Prescribe carbamazepine (Tegretol) 250mg PR STAT
- +/- Prescribe digoxin 100micrgrams IV STAT
- +/- Prescribe sliding scale as per local guidelines

**Debrief**

Return to intended learning outcomes:

- Demonstrate team working skills and understand the different roles within the team
- Demonstrate appropriate communication skills with members of the multidisciplinary team
- Demonstrate an understanding of the immediate management of acute stroke
- Demonstrate an understanding of medication rationalisation in patient who is nil by mouth

| **Patient Name CHI Date of Birth Age**  Andrew Robertson 010147XXXX 01/01/1947 74 | | | | | | | | | |
| --- | --- | --- | --- | --- | --- | --- | --- | --- | --- |
| **Source of Information** | | | | | | | | | |
| Patient  Care home / MAR Chart |  | Relative/Carer  Previous Discharge Letter |  | Patients Own Drugs  Repeat Prescription Slip |  | GP letter  Community Pharmacy |  | GP Practice  Other (Please state) |  |
|  |  |  |  |  |  |  |  |  |  |

| Allergy Description | Date Recorded | Comments |
| --- | --- | --- |
| Penicillin | 01/03/1999 | Anaphylaxis |

| **Acute Medication (within 30 days)** | | | | | | | | | | |
| --- | --- | --- | --- | --- | --- | --- | --- | --- | --- | --- |
| Originator | Drug ID | Formulation | Dose | Frequency | Medication Start Date | Prescription Date | Continue | Withhold | Stop | Comments |
| In Practice | Ibuprofen | 200 mg Tablets | TWO TO BE TAKEN THREE TIMES A DAY |  | 10 days ago | 10 days ago |  |  |  |  |
| In Practice | Cetirizine | 10 mg Tablets | ONE TO BE TAKEN DAILY |  | 7 days ago | 7 day ago |  |  |  |  |
|  |  |  |  |  |  |  |  |  |  |  |
|  |  |  |  |  |  |  |  |  |  |  |
|  |  |  |  |  |  |  |  |  |  |  |

| **Repeat Medication** | | | | | | | | | | |
| --- | --- | --- | --- | --- | --- | --- | --- | --- | --- | --- |
| Originator | Drug ID | Formulation | Dose | Frequency | Medication Start Date | Prescription Date | Continue | Withhold | Stop | Comments |
|  | Warfarin | 1 mg Tablets | AS DIRECTED |  | 02/03/2001 | 1 month ago |  |  |  |  |
|  | Warfarin | 3 mg Tablets | AS DIRECTED |  | 02/03/2001 | 1 month ago |  |  |  |  |
|  | Morphine Sulfate | 10 mg / 5 ml Oral Solution | 2.5MLS FOR BREAKTHROUGH PAIN UPTO MAXIMUM 4 TIMES PER DAY |  | 27/8/2017 | 1 month ago |  |  |  |  |
|  | Levetiracetam | 500 mg Tablets | ONE TO BE TAKEN TWICE A DAY |  | 09/07/2003 | 1 month ago |  |  |  |  |
|  | Levetiracetam | 250 mg Tablets | ONE TO BE TAKEN TWICE A DAY |  | 10/01/2007 | 1 month ago |  |  |  |  |
|  | Carbamazepine (Tegretol) | 200 mg Tablets | ONE TO BE TAKEN TWICE DAILY |  | 14/4/2008 | 1 month ago |  |  |  |  |
|  | Digoxin | 125 microgram Tablets | ONE TO BE TAKEN EACH DAY |  | 02/03/2001 | 1 month ago |  |  |  |  |

| Compliance Device Name and telephone number for community pharmacy | | | | | |
| --- | --- | --- | --- | --- | --- |
|  |  | | | | |
| Completed by Designation Grade Date Time Contact Number | | | | | |
|  |  |  |  |  |  |
| Reviewed by Designation Grade Date Time Contact Number | | | | | |
|  |  |  |  |  |  |
