# Supplementary material for: Exploring transformative learning for trainee pharmacists through interprofessional simulation: a constructivist interview study
Source: Adv Simul (Lond). 2021 Sep 7;6:31. doi: 10.1186/s41077-021-00180-2 (PMC8422059; doi:10.1186/s41077-021-00180-2)
Supplement: Supplementary file 3 — Additional file 3. [file 41077_2021_180_MOESM3_ESM.docx]

**Asthma Exacerbation in a Young Male**

**OUTLINE OF SESSION**

**Target Learners**

5^th^ Year Medical Student & Pre-registration/Junior Pharmacist.

**Synopsis**

Laurence Nicol, a 20 year old male, is brought in by ambulance. He is wheezy and short of breath.

**Learning Objectives**

- Demonstrate team working skills and understand the different roles within the team
- Demonstrate appropriate communication skills with members of the multidisciplinary team
- Demonstrate a structured approach to commencing treatment for asthma exacerbation

**Environment, equipment, essential props**

- Sim room
- 2+ faculty members
- ED Yellow sheet
- Emergency Care Summary (Medicines Reconciliation Report)
- High risk infusion chart
- Laptop (for access to NICE Asthma guidelines / BNF/ Medusa)
- ECG result
- CXR result
- ABG result
- Blood results
- NEWS chart

**Patient set up**

In bed monitoring attached.

**Other participants**

Staff nurse.

**SCENARIO SCRIPT**

**Background information**

Known asthma.

**Guidance for faculty (key aims)**

- Recognise asthma attack and treat appropriately according to severity
- Recognise deteriorating patient and respond appropriately with reassessment and treatment escalation
- Recognise and investigate possible reasons for asthma attack
- MDT working

**Predicted derails** (things which might not go according to plan)

- Does not identify asthma attack and assess severity – nurse prompt to NICE guidelines
- Does not treat asthma appropriately according to severity – nurse prompt to NICE guidelines
- Does not recognise patient deterioration – nurse/patient prompt
- Does not act appropriately to patient deterioration – nurse prompt to NICE guidelines
- Does not recognise poor compliance with medication – patient prompt

**Optional add-ins**

Discuss escalation plan if further deterioration – senior / anaesthetics support and possible need for ITU admission.

| State | Events | Desired learner behaviours and triggers to move to next state | | | |
| --- | --- | --- | --- | --- | --- |
|  |  | Medic actions | Pharmacist actions | Mannequin operator | Teaching points |
| 1 | A: maintained  B: able to complete sentences, sats 95%, RR 24, widespread wheeze.  C: HR 100. BP 122/80  D: Temp 36.1. BM 6  E: Abdo SNT. | ABCDE assessment.  Request appropriate investigations: CXR, ECG, bloods  Gain IV access  Initiates management: salbutamol/ipratropium nebs; steroids | Complete medicines reconciliation (confirm with patient)  Advise on the appropriate initial management of asthma attack  Review prescriptions | Breathless patient but able to complete sentences.  ECG: sinus tachy.  CXR: no acute findings | Conduct an ABCDE assessment  Initial asthma management. |
| 2 | A: maintained  B: struggling to complete sentences, sats 93%. RR 27, widespread wheeze.  C: HR 110. BP 122/80 | Reassess.  Further nebs.  ABG.  Ask for help.  Commence Magnesium sulphate.  Prescribe oxygen | Advise on the use of magnesium sulphate  Assist with prescribing on high risk chart | Unable to complete sentences.  ABG. | Recognise a deteriorating patient and ask for help.  Interpret ABG.  Prescribe magnesium appropriately and work together to do so. |
| 3 | A: maintained  B: able to complete sentences, sats 95%, RR24, widespread wheeze.  C: HR 100. BP 122/80 | Reassess.  Recognise improvement and need for admission to hospital. | Discuss with patient about non-compliance & reasons for this  Educate patient on use of preventer medication  Educate patient on avoidance of allergens | Patient begins to be able to talk again and able to give history |  |

**Results**

Bloods: no significant findings

CXR: clear lung fields, no consolidation, no pneumothorax.

ECG: sinus tachycardia

| ARTERIAL BLOOD GAS  pO_2_ 10.1  pCO_2_ 5  H^+^ 40  HCO_3_^-^ 22  Lac 1.1.  Normal values:  pO_2_ 11.3-12.6  pCO_2_ 4.7 -6  H^+^ 35-45  HCO_3_^-^ 21-29  Lac 0.5-1.6 |
| --- |

**Expected Prescription**

- Salbutamol 2.5-5mg nebule (oxygen driven when reaches State 2)
- +/- Ipratropium 500microgram nebule (oxygen driven when reaches State 2)
- Prednisolone 40-50mg STAT or intravenous hydrocortisone sodium succinate 100mg STAT
- Intravenous magnesium sulphate 1.2-2g infusion over 20 minutes

**Debrief**

Return to intended learning outcomes:

- Demonstrate team working skills and understand the different roles within the team
- Demonstrate appropriate communication skills with members of the multidisciplinary team
- Demonstrate a structured approach to commencing treatment for asthma exacerbation

| **Patient Name CHI Date of Birth Age**  Laurence Nicol 251000XXXX 25/10/2000 20 | | | | | | | | | |
| --- | --- | --- | --- | --- | --- | --- | --- | --- | --- |
| **Source of Information** | | | | | | | | | |
| Patient  Care home / MAR Chart |  | Relative / Carer  Previous Discharge Letter |  | Patient’s Own Drugs  Repeat Prescription Slip |  | GP letter  Community Pharmacy |  | GP Practice  Other (Please state) |  |
|  |  |  |  |  |  |  |  |  |  |

| Allergy Description | Date Recorded | Comments |
| --- | --- | --- |
|  |  |  |

| **Acute Medication (within 30 days)** | | | | | | | | | | |
| --- | --- | --- | --- | --- | --- | --- | --- | --- | --- | --- |
| Originator | Drug ID | Formulation | Dose | Frequency | Medication Start Date | Prescription Date | Continue | Withhold | Stop | Comments |
|  |  |  |  |  |  |  |  |  |  |  |

| **Repeat Medication** | | | | | | | | | | |
| --- | --- | --- | --- | --- | --- | --- | --- | --- | --- | --- |
| Originator | Drug ID | Formulation | Dose | Frequency | Medication Start Date | Prescription Date | Continue | Withhold | Stop | Comments |
|  | Salbutamol | 100microgram CFC-Free metered dose Inhaler | 2 PUFFS TO BE TAKEN WHEN REQUIRED |  | 05/03/2017 | 6 months ago |  |  |  |  |
|  | Clenil Modulite | 100microgram CFC-Free metered dose inhaler | TWO PUFFS TO BE TAKEN TWICE A DAY |  | 05/03/2017 | 6 months ago |  |  |  |  |
|  |  |  |  |  |  |  |  |  |  |  |
|  |  |  |  |  |  |  |  |  |  |  |
|  |  |  |  |  |  |  |  |  |  |  |

| Compliance Device Name and telephone number for community pharmacy | | | | | |
| --- | --- | --- | --- | --- | --- |
|  |  | | | | |
| Completed by Designation Grade Date Time Contact Number | | | | | |
|  |  |  |  |  |  |
| Reviewed by Designation Grade Date Time Contact Number | | | | | |
|  |  |  |  |  |  |
