# Supplementary material for: Exploring transformative learning for trainee pharmacists through interprofessional simulation: a constructivist interview study
Source: Adv Simul (Lond). 2021 Sep 7;6:31. doi: 10.1186/s41077-021-00180-2 (PMC8422059; doi:10.1186/s41077-021-00180-2)
Supplement: Supplementary file 4 — Additional file 4. [file 41077_2021_180_MOESM4_ESM.docx]

**Urosepsis in Parkinson’s Disease**

**OUTLINE OF SESSION**

**Target Learners**

5^th^ Year Medical Student & Pre-registration/Junior Pharmacist.

**Synopsis**

Charles McPartland, a 74 year old male, is brought in by ambulance ‘unwell’. Family member is following behind.

**Learning Objectives**

- Demonstrate team working skills and understand the different roles within the team
- Demonstrate appropriate communication skills with members of the multidisciplinary team
- Demonstrate a structured approach to managing sepsis
- Demonstrate a structured approach to managing Parkinson’s when patient nil by mouth

**Environment, equipment, essential props**

- Sim room
- 2+ faculty members
- ED Yellow sheet
- Emergency Care Summary (Medicines Reconciliation Report)
- Gentamicin chart
- Fluid prescription chart
- Laptop (for access to Antimicrobial Companion / Parkinson’s guidelines / BNF / Parkinson’s UK Website)
- ECG result
- VBG result
- Urinalysis
- NEWS Chart

**Patient set up**

In bed monitoring attached.

**Other participants**

Staff nurse, family member, senior support (over the phone if needed).

**SCENARIO SCRIPT**

**Background information**

Can’t find ambulance handover, Clinical Portal has crashed.

**Guidance for faculty (key aims)**

- Recognise and manage sepsis
- Management of Parkinson’s medication
- MDT working

**Predicted derails** (things which might not go according to plan)

- Does not identify sepsis/forgets sepsis 6 – prompt from nurse to call for senior support
- Does not take full collateral from family – family prompt
- Does not recognise need for Parkinson’s medicine patch – senior prompt
- Requests NG tube – nurse advises none available
- Mistakes situation for other Parkinson’s complication such as severe dopamine agonist withdrawal (Neuroleptic malignant like syndrome), uncontrolled dyskinesia, psychosis, aspiration pneumonia – not hyperthermic, no respiratory signs, nurse prompt.

**Optional add-ins**

- Antiemetic (domperidone best, also can give cyclizine/ondansetron. Not metoclopramide)
- Analgesia (need to consider swallowing difficulties)

| State | Events | Desired learner behaviours and triggers to move to next state | | | |
| --- | --- | --- | --- | --- | --- |
|  |  | Medic actions | Pharmacist actions | Mannequin operator | Teaching points |
| 1 | A: maintained  B: RR 14, O_2_ 98%  C: HR 110, BP 96/58 | ABCDE assessment.  IV access, bloods (gas and cultures), fluids  +/- calls for help | Complete medicines reconciliation | Patient unable to give history  ECG sinus tachycardia | Don’t move on until stabilised    Task delegation |
| 2 | A: maintained  B: RR 14, O_2_ 98%  C: HR 89, BP 110/70  D: GCS 12 (E3, V3, M6)  BM 6  E: Temp 38.1^o^C. Tender suprapubic region | Requests urine MC&S +/- catheter  +/- requests CXR  Diagnose urosepsis  Antibiotics as per antimicrobial guidelines | Gentamicin prescription  Dosage calculation given no recent bloods | Eyes open to voice, incomprehensible words | SIRS criteria Sepsis 6  Team working, task delegation    Gentamicin prescription |
| 3 | A: maintained  B: RR 14, O_2_ 98%  C: HR 89, BP 110/70  D: GCS 12 (E3, V3, M6)  BM 6  E: Temp 38.1^o^C. Tender suprapubic region | Reassess.  VBG and ECG available  (bloods not back yet , no CXR)  Make senior aware |  | Senior not available | Reassess  Metabolic acidosis on VBG |
| 4 | Family arrive – HPC has been confused for two days, urinary frequency. Concerned that patient is more rigid and less communicative. PMH Parkinson’s, good cognitive baseline. DH NKDA, as per ECS. SH Last seen 2 days ago. OD carer. ?taken Parkinson’s meds. | Collateral history  Recognition of need to give Parkinson’s medicines.  Consider NG tube (none available)  Prescribes rotigotine patch | Co-careldopa and dopamine agonist conversion to Rotigotine patch  +/- advice on antiemetic and analgesia | +/- patient feels nauseous, pain | Oral to patch for Parkinson’s medications  +/- antiemetic in Parkinson’s |

**Results**

VBG: metabolic acidosis with mildly raised lactate

Urinalysis: positive for leukocytes, positive for nitrates

Bloods: none available

CXR: none available

ECG: sinus tachycardia

| VENOUS BLOOD GAS  pH 7.28  pO_2_ 7.1  pCO_2_ 4.0  H^+^ 48  HCO_3_^-^ 18  Lac 4.0  Normal values:  pH 7.35-7.45  pO_2_ 11.3-12.6  pCO_2_ 4.7 -6  H^+^ 35-45  HCO_3_^-^ 21-29  Lac 0.5-1.6 |
| --- |

**Expected Prescription**

Gentamicin:

Step 1: Calculate the does using the manual calculation if blood results (creatinine) not available

5mg/kg (maximum 400mg) or if CKD 5, give 2.5mg/kg (maximum 180mg) and liaise with senior staff.

Calculate the dosage regime using the online gentamicin calculator when bloods available.

Step 2: Advise on monitoring of gentamicin concentration; 6-14hours post dose after the start of the

first gentamicin infusion or after 24 hours if CrCl<21ml/min.

Parkinson’s management:

Co-careldopa 125mg (25/100) three times daily and pramipexole (domaine agonist) 88micrograms three times daily – switch to rotigotine 8mg/24hr patch as per hospital guideline. Refer patient to Parkinson’s disease specialist.

**Debrief**

Return to intended learning outcomes:

- Demonstrate team working skills and understand the different roles within the team
- Demonstrate appropriate communication skills with members of the multidisciplinary team
- Demonstrate a structured approach to managing sepsis
- Demonstrate a structured approach to managing Parkinson’s when patient nil by mouth

| **Patient Name CHI Date of Birth Age**  Charles McPartland 060547XXXX 06/05/1947 74 | | | | | | | | | |
| --- | --- | --- | --- | --- | --- | --- | --- | --- | --- |
| **Source of Information** | | | | | | | | | |
| Patient  Care home / MAR Chart |  | Relative / Carer  Previous Discharge Letter |  | Patient’s Own Drugs  Repeat Prescription Slip |  | GP letter  Community Pharmacy |  | GP Practice  Other (Please state) |  |
|  |  |  |  |  |  |  |  |  |  |

| Allergy Description | Date Recorded | Comments |
| --- | --- | --- |
| Ibuprofen | 09/11/1998 | GI upset |

| **Acute Medication (within 30 days)** | | | | | | | | | | |
| --- | --- | --- | --- | --- | --- | --- | --- | --- | --- | --- |
| Originator | Drug ID | Formulation | Dose | Frequency | Medication Start Date | Prescription Date | Continue | Withhold | Stop | Comments |
| In Practice | Trimethoprim | 200mg Tablets | ONE TO BE TAKEN TWICE A DAY |  | 5 days ago | 5 days ago |  |  |  |  |
|  |  |  |  |  |  |  |  |  |  |  |
| In Practice | Co-codamol | 30 mg/500 mg Tablets | ONE OR TWO TO BE TAKEN FOUR TIMES A DAY WHEN REQUIRED FOR PAIN |  | 5 days ago | 5 days ago |  |  |  |  |

| **Repeat Medication** | | | | | | | | | | |
| --- | --- | --- | --- | --- | --- | --- | --- | --- | --- | --- |
| Originator | Drug ID | Formulation | Dose | Frequency | Medication Start Date | Prescription Date | Continue | Withhold | Stop | Comments |
|  | Co-careldopa | 25 mg/100 mg  Tablets | ONE TO BE TAKEN THREE TIMES A DAY |  | 01/05/2008 | 2 weeks ago |  |  |  |  |
|  | Pramipexole | 88 microgram Tablets | ONE TO BE TAKEN THREE TIMES A DAY |  | 01/05/2008 | 2 weeks ago |  |  |  |  |
|  | Zopiclone | 3.75 mg Tablets | ONE TO BE TAKEN AT NIGHT |  | 01/05/2017 | 3 months ago |  |  |  |  |
